# Real-world outcomes of avacopan beyond the first year in antineutrophil cytoplasmic antibody-associated vasculitis: a retrospective cohort study

**DOI:** 10.1186/s41927-026-00655-7

**Published:** 2026-05-19

**Authors:** Makoto Yamaguchi, Hirokazu Sugiyama, Hiroshi Kinashi, Kentaro Imai, Keisuke Kamiya, Takayuki Katsuno, Takahiro Imaizumi, Shogo Banno, Yasuhiko Ito, Takuji Ishimoto

**Affiliations:** 1 Department of Nephrology and Rheumatology, Aichi Medical University, 1-1 Karimata, Yazako, Nagakute, Aichi 480-1195 Japan; 2 Department of Nephrology and Rheumatology, Aichi Medical University Medical Center, Okazaki, Aichi Japan; 3 Data Coordinating Center, Department of Advanced Medicine, Nagoya University Hospital, Nagoya, Japan

**Keywords:** Antineutrophil cytoplasmic autoantibody-associated vasculitis, Avacopan, Efficacy, Glucocorticoid-sparing therapy, Real-world evidence, Remission, Safety

## Abstract

**Background:**

Avacopan is effective in ANCA-associated vasculitis (AAV) in randomized trials, and real-world studies have supported its clinical effectiveness. However, treatment-emergent adverse events (TEAEs) often lead to discontinuation, and data beyond 1 year remain limited.

**Methods:**

We retrospectively studied 40 adults with newly diagnosed or relapsing AAV treated with avacopan. Patients were classified into continuation (*n* = 23) and discontinuation (*n* = 17) groups according to discontinuation due to avacopan-related TEAEs.

**Results:**

During a median follow-up of 29 months (interquartile range 26–32), avacopan-related TEAEs occurred in 21 patients (52.5%) at a median of 1.4 months following initiation; all patients in the discontinuation group stopped avacopan because of TEAEs. Clinical remission (BVAS = 0 and no glucocorticoid use) was achieved in 47.5%, 62.5%, and 87.2% of patients at 6, 12, and 24 months, respectively. Five patients (21.7%) in the continuation group remained in remission on avacopan monotherapy for a median of 24 months after withdrawing all other immunosuppressive agents.

**Conclusions:**

In this retrospective single-center cohort, clinical outcomes beyond 1 year appeared broadly comparable between patients who continued avacopan and those who discontinued it early because of TEAEs. Further prospective studies are needed to define the optimal use, patient selection, and duration of avacopan therapy in AAV.

**Clinical trial number:**

Not applicable.

**Supplementary Information:**

The online version contains supplementary material available at 10.1186/s41927-026-00655-7.

## Background

Antineutrophil cytoplasmic antibody (ANCA)-associated vasculitis (AAV) is a systemic form of small-vessel vasculitis characterized by neutrophil activation, endothelial injury, and recurrent relapses [1, 2]. Activation of the complement system, particularly C5a–complement 5a receptor 1 (C5aR1) signaling, plays a key pathogenic role in AAV by promoting neutrophil priming, neutrophil extracellular trap (NET) formation, and vascular injury [3–6]. Although induction therapy with glucocorticoids and rituximab (RTX) has improved outcomes, long-term disease control remains challenging due to treatment-related toxicity and the persistent risk of relapse [7].

Avacopan, an oral C5aR1 inhibitor, targets this effector pathway and demonstrated efficacy in achieving sustained remission and reducing glucocorticoid exposure in the ADVOCATE trial [8, 9]. In addition, several real-world studies have supported the clinical effectiveness of avacopan [10–15]. A recent systematic review focusing exclusively on real-world avacopan studies, including our previous report [10], reported high 6-month remission rates, while also noting that hepatotoxicity among treatment-emergent adverse events (TEAEs) appeared to be more frequently reported in Japanese cohorts [16]. In routine practice, TEAEs, particularly liver enzyme elevations, may lead to avacopan discontinuation, and severe hepatotoxicity has also been reported [17, 18].

Despite accumulating real-world evidence, data beyond the first 12 months of therapy remain limited, leaving uncertainty regarding the long-term effectiveness, safety, and treatment persistence of avacopan in routine care. In our previous report [10], early discontinuation due to adverse events was relatively common; however, short-term outcomes within 12 months were broadly comparable between patients who continued avacopan and those who discontinued treatment. Whether these findings extend beyond the first year remains unclear.

Building on our earlier findings [10], we aimed to evaluate the real-world outcomes of avacopan beyond 1 year after treatment initiation, focusing on clinical effectiveness, safety, treatment persistence, treatment patterns, and factors associated with continuation or discontinuation. This study may help clarify the role of avacopan in real-world AAV management.

## Methods

### Patients

This retrospective study included 40 consecutive adult patients with newly diagnosed or relapsed AAV who received avacopan as part of induction therapy between October 2021 and January 2024 at the Nephrology and Rheumatology Centers of Aichi Medical University, Japan. AAV was diagnosed according to the 2012 revised Chapel Hill Consensus Conference definitions and further classified as granulomatosis with polyangiitis or microscopic polyangiitis (MPA) using the European Medicines Agency vasculitis classification algorithm [19].

This study was approved by the Ethics Committee of Aichi Medical University (approval no. 2019-H151) and conducted in accordance with the Declaration of Helsinki. Informed consent was obtained from all patients.

### Study variables

Study variables included baseline demographic characteristics (age, sex, and body mass index), disease-related variables (AAV subtype, disease status, Birmingham Vasculitis Activity Score [BVAS] 2003 [20], organ involvement, and ANCA serostatus), renal parameters, including baseline estimated glomerular filtration rate (eGFR), calculated using the Japanese equation: eGFR (mL/min/1.73 m²) = 194 × Scr^−1.094 × age^−0.287 × 0.739 if female [21], and proteinuria, inflammatory markers (C-reactive protein), treatment-related factors (induction regimen, rituximab use, glucocorticoid dose, and time to avacopan initiation), and the occurrence of TEAEs. As previously described [10], baseline clinical characteristics and treatment data were collected at the initiation of immunosuppressive therapy in patients with newly diagnosed or relapsed AAV. Owing to the retrospective nature of this study, treatment protocols were not predetermined and were left to the discretion of the treating nephrologists. During the study period, except for one patient who declined avacopan treatment, avacopan was consecutively administered to all patients diagnosed with MPA or GPA. In principle, avacopan was continued beyond 12 months unless treatment-emergent adverse events occurred, with the aim of reducing concomitant immunosuppressive therapy as much as possible. The choice and tapering of concomitant immunosuppressive agents were individualized according to disease severity, organ involvement, prior treatment exposure, comorbidities, and physician judgment. Details of immunosuppressive therapies were recorded, including rituximab, azathioprine, methylprednisolone pulse therapy (0.5–1.0 g/day for three consecutive days), mizoribine, an inosine monophosphate dehydrogenase inhibitor used in Japan as an immunosuppressive agent, and mycophenolate mofetil. Rituximab was used for both induction and maintenance therapy. For remission induction, rituximab was administered at a dose of 375 mg/m², usually as a single infusion, although some patients received two infusions at the discretion of the treating physicians. Because our cohort included many older patients, a single infusion was frequently selected in consideration of infection risk. For remission maintenance, rituximab was generally re-administered at the same dose every 6 months. Glucocorticoid use, including the daily prednisolone dose (mg/day) at 1, 3, 6, 12, and 24 months after initiation of induction therapy, was also recorded. Liver function monitoring was performed at the discretion of the treating physicians; however, as a general practice at our institution, serum liver enzymes were assessed at least weekly during the first two months after avacopan initiation, at least every two weeks between two and three months, and at least monthly thereafter. Elevated liver enzymes were defined as aspartate aminotransferase (AST) and/or alanine aminotransferase (ALT) levels > 3× the upper limit of normal. The severity of liver enzyme elevation was graded according to the Common Terminology Criteria for Adverse Events (CTCAE) version 5.0. In all patients with liver enzyme elevation, hepatobiliary imaging showed no abnormalities, and viral causes were excluded based on serological and virological testing. These variables were compared between the continuation and discontinuation groups to explore potential predictors of avacopan discontinuation.

### Outcomes

The primary outcomes were the proportions of patients achieving clinical remission at 6, 12, and 24 months. Clinical remission was defined as a BVAS of 0 and no glucocorticoid use [8, 9]. Disease relapse was defined as recurrence of AAV requiring escalation of immunosuppressive therapy at any stage of treatment [22]. Additional outcomes included mortality, progression to end-stage kidney disease (ESKD) requiring dialysis, hospitalization due to infection, and changes in eGFR at 12 and 24 months in patients with renal involvement.

As previously reported [10], safety was assessed by recording treatment-emergent adverse events (TEAEs) associated with avacopan. Causality was determined by the treating physicians on the basis of temporal association, exclusion of alternative causes, and response to dose reduction or discontinuation.

The observation period was defined as the interval from the initiation of immunosuppressive therapy to the last hospital visit. Patients were followed through January 31, 2026, and data were censored at the last hospital visit before that date. Thirty-nine patients (97.5%) completed at least 24 months of follow-up, whereas one patient was censored at 14.8 months after the initiation of immunosuppressive therapy because the patient was transferred to another hospital before January 31, 2026.

### Statistical analysis

Clinical characteristics were compared between patients who discontinued avacopan because of TEAEs and those who continued treatment using the Wilcoxon rank-sum test or Fisher’s exact test, as appropriate. Statistical significance was defined as *P* < 0.05. All statistical analyses were performed using JMP version 14.0.0 (SAS Institute, Cary, NC) and Stata version 13.0 (StataCorp LP, College Station, TX).

## Results

### Study participants and clinical characteristics

The baseline demographic and clinical characteristics of the continuation and discontinuation groups are summarized in Table 1. A total of 40 patients with AAV were included in the analysis. Of these, 23 patients continued avacopan (continuation group), whereas 17 discontinued avacopan (discontinuation group). The median age of the overall cohort was 78 years (interquartile range [IQR] 70–80), and 52.5% of patients were male. No significant differences were observed between groups in age, sex, body mass index, or baseline renal function (*P* > 0.05). The median baseline eGFR was 45 mL/min/1.73 m², and 9/40 patients (22.5%) had an eGFR < 15 mL/min/1.73 m²; one patient (2.5%) was on chronic dialysis at baseline. MPA was the predominant AAV subtype (87.5%), with a smaller proportion of granulomatosis with polyangiitis (12.5%). 70% of patients had newly diagnosed disease and 30% had relapsing disease, with similar distributions between the two groups. Myeloperoxidase-ANCA positivity was observed in 85.0% and proteinase 3 (PR3)-ANCA positivity in 12.5%; ANCA serostatus did not differ significantly between groups. The median baseline BVAS was 16 in both groups.

**Table 1:** Comparison of baseline characteristics between avacopan continuation and discontinuation groups

	All (*n* = 40)	Continuation (*n* = 23)	Discontinuation (*n* = 17)	*P*-value
Age, year	78 (70–80)	77 (61–80)	78 (75–80)	0.394
Male sex	21 (52.5%)	11 (47.8%)	10 (58.8%)	0.491
Body weight (kg)	52 (48–62)	52 (48–61)	55 (46–64)	0.612
BMI, kg/m^2^	21 (19–23)	20.4 (18.6–22.3)	21.1 (18.7–23.8)	0.395
eGFR, mL/min/1.73 m^2^	45 (21–59)	44 (24–70)	49 (16–59)	0.640
eGFR < 15 mL/min/1.73 m^2^	9 (22.5%)	5 (21.7%)	4 (23.5%)	0.893
Chronic dialysis	1 (2.5%)	1 (4.3%)	0	0.250
Serum albumin, g/dL	2.7 (2.1–3.6)	2.6 (2.3–3.8)	2.7 (1.9–3.0)	0.298
CRP level, mg/dL	6.0 (1.5–11.0)	4.2 (1.4–10.9)	6.1 (4.3–12.2)	0.404
Type of ANCA-associated vasculitis				0.904
GPA	5 (12.5%)	3 (13.0%)	2 (11.8%)	
MPA	35 (87.5%)	20 (87.0%)	15 (88.2%)	
ANCA-associated vasculitis status				0.530
Newly diagnosed	28 (70.0%)	17 (73.9%)	11 (64.7%)	
Relapsed	12 (30.0%)	6 (26.1%)	6 (35.3%)	
ANCA positivity				0.880
PR3-ANCA positive	5 (12.5%)	3 (13.0%)	2 (11.8%)	
MPO-ANCA positive	34 (85.0%)	20 (87.0%)	14 (82.3%)	
ANCA-negative	1 (2.5%)	0	1 (5.9%)	
BVAS	16 (12–18)	16 (12–18)	16 (12–20)	0.542
Organ involvement				
Constitutional	37 (92.5%)	21 (91.3%)	16 (94.1%)	0.738
Cutaneous	2 (5.0%)	2 (8.7%)	0	0.212
Ear, nose, and throat	6 (15.0%)	4 (17.4%)	2 (11.8%)	0.622
Pulmonary	22 (55.0%)	13 (56.5%)	9 (52.9%)	0.738
Diffuse alveolar hemorrhage	3 (7.5%)	1 (4.3%)	2 (11.8%)	
Interstitial lung disease	15 (37.5%)	10 (43.5%)	5 (29.4%)	
Nodules	2 (5.0%)	1 (4.3%)	1 (5.9%)	
Pleuritis	1 (2.5%)	0	1 (5.9%)	
Heart	0	0	0	
Abdominal	0	0	0	
Nervous system	10 (25.0%)	6 (26.1%)	4 (23.5%)	0.854
Kidney	25 (62.5%)	15 (65.2%)	10 (58.8%)	0.546
Hematuria	25 (62.5%)	15 (65.2%)	10 (58.8%)	0.546
Proteinuria, g/gCr	1.0 (0.1–1.6)	1.1 (0.2–1.6)	0.5 (0.1–1.6)	0.342

Data are presented as numbers (%) or medians (interquartile ranges)

**Abbreviations**: BMI, body mass index; eGFR, estimated glomerular filtration rate; CRP, C-reactive protein; ANCA, antineutrophil cytoplasmic autoantibody; GPA, granulomatosis with polyangiitis; MPA, microscopic polyangiitis; BVAS, Birmingham Vasculitis Activity Score; PR3, anti-proteinase 3; MPO, anti-myeloperoxidase

Regarding organ involvement, constitutional symptoms were present in 92.5% of patients, pulmonary involvement in 55.0%, and renal involvement in 62.5%. The frequencies of diffuse alveolar hemorrhage, interstitial lung disease, neurological involvement, and renal manifestations (hematuria and proteinuria) were comparable between the continuation and discontinuation groups.

### Immunosuppressive treatment and glucocorticoid tapering

Details of the induction and maintenance immunosuppressive therapies and glucocorticoid tapering are shown in Table 2. The median interval from induction therapy initiation to avacopan introduction was 12 days and did not differ significantly between the continuation and discontinuation groups (13 vs. 9 days, *P* = 0.223). In contrast, the duration of avacopan use differed markedly between the two groups, with a median of 28 months in the continuation group and 1.3 months in the discontinuation group. RTX was the primary induction agent and was administered to 37/40 patients (92.5%), with similar proportions in the continuation and discontinuation groups (21/23 [91.3%] vs. 16/17 [94.2%], *P* = 0.673). Cyclophosphamide was not used in this cohort, reflecting physician preference to avoid cyclophosphamide-related toxicity in this predominantly older population with comorbidities. Among the 37 patients who received rituximab for remission induction, rituximab was administered as a single infusion of 375 mg/m² in 19/21 patients (90.5%) in the continuation group and 15/16 patients (93.8%) in the discontinuation group, whereas two infusions were given in 2/21 patients (9.5%) and 1/16 patients (6.3%), respectively. The use of methylprednisolone pulse therapy was also comparable between the groups (30.4% vs. 47.1%, *P* = 0.283). For maintenance therapy, RTX was administered to 77.5% of patients overall, and other conventional immunosuppressive agents (azathioprine, mizoribine, and mycophenolate mofetil) were used infrequently. Avacopan monotherapy at the last follow-up was observed only in the continuation group (5/23 [21.7%]), after withdrawal of glucocorticoids and all concomitant non-glucocorticoid immunosuppressive agents.

**Table 2:** Comparison of immunosuppressive treatment between avacopan continuation and discontinuation groups

	All (*n* = 40)	Continuation (*n* = 23)	Discontinuation (*n* = 17)	*P*-value
**Treatment**				
Time to start avacopan, days	12 (6–24)	13 (7–26)	9 (6–15)	0.223
Induction therapy				0.673
Rituximab	37 (92.5%)	21 (91.3%)	16 (94.2%)	
Azathioprine	2 (5.0%)	1 (4.3%)	1 (5.9%)	
GC monotherapy	1 (2.5%)	1 (4.3%)	0	
Use of methylprednisolone pulse therapy	15 (37.5%)	7 (30.4%)	8 (47.1%)	0.283
**Maintenance therapy**				0.162
Rituximab	31 (77.5%)	17 (73.9%)	14 (82.4%)	
Azathioprine	2 (5.0%)	1 (4.3%)	1 (5.9%)	
Mizoribine	1 (2.5%)	0	1 (5.9%)	
Mycophenolate mofetil	1 (2.5%)	0	1 (5.9%)	
**Avacopan monotherapy at last follow-up**	5 (12.5%)	5 (21.7%)	0	
**Prednisolone dose**				
Initial dose, mg/day (*n* = 40)	40 (30–58)	40 (30–50)	40 (30–60)	0.933
Dose at 1 month, mg/day (*n* = 40)	10 (5–10)	10 (5–10)	10 (5–10)	0.811
Dose at 3 months, mg/day (*n* = 40)	5 (2.5–5)	4 (1–5)	5 (4–5)	0.070
Dose at 6 months, mg/day (*n* = 40)	2 (0–4)	0 (0–3)	3 (0–5)	0.392
Dose at 12 months, mg/day (*n* = 40)	0 (0–2)	0 (0–1)	0 (0–2)	0.319
Dose at 24 months, mg/day (*n* = 39)	0	0	0	0.941
Off prednisolone at 1 month (*n* = 40)	1 (2.5%)	0	1 (5.9%)	0.239
Off prednisolone at 3 months (*n* = 40)	7 (17.5%)	5 (21.7%)	2 (11.8%)	0.412
Off prednisolone at 6 months (*n* = 40)	19 (47.5%)	12 (52.2%)	7 (41.2%)	0.491
Off prednisolone at 12 months (*n* = 40)	25 (62.5%)	16 (69.6%)	9 (52.9%)	0.283
Off prednisolone at 24 months (*n* = 39)	34 (87.2%)	20 (87.0%)	14 (87.5%)	0.960
Off prednisolone at last follow-up (*n* = 40)	34 (85.0%)	20 (87.0%)	14 (82.4%)	0.687
Time to stop prednisolone, months (*n* = 34)	5.8 (4.0–12.9)	5.6 (3.0–12.4)	7.2 (4.6–13.9)	0.495

Data are presented as numbers (%) or medians (interquartile ranges)

Avacopan monotherapy at last follow-up was defined as maintenance of remission with avacopan alone after withdrawal of glucocorticoids and all concomitant non-glucocorticoid immunosuppressive agents, including rituximab and conventional immunosuppressive agents. This represents the final treatment status and does not imply absence of prior induction or maintenance therapy

The median initial oral prednisolone dose was 40 mg/day and did not differ between groups. Prednisolone doses at 1, 3, 6, 12, and 24 months, as well as the median time to complete glucocorticoid withdrawal, were similar between groups. At 6, 12, and 24 months, 47.5%, 62.5%, and 87.2% of patients had discontinued glucocorticoids, respectively, with no significant between-group differences.

### Clinical outcomes

Clinical outcomes, including remission rates, relapse, renal outcomes, and infections, are summarized in Table 3. Clinical remission was achieved in 47.5%, 62.5%, and 87.2% of patients at 6, 12, and 24 months, respectively, with comparable rates in the continuation and discontinuation groups at each time point. Because BVAS was 0 in all evaluable patients at 6, 12, and 24 months, remission rates numerically paralleled glucocorticoid-free status at these time points.

**Table 3:** Comparison of outcomes between avacopan continuation and discontinuation groups

	All (*n* = 40)	Continuation (*n* = 23)	Discontinuation (*n* = 17)	*P*-value
Clinical remission at 6 months (*n* = 40)	19 (47.5%)	12 (52.2%)	7 (41.2%)	0.491
Clinical remission at 12 months (*n* = 40)	25 (62.5%)	16 (69.6%)	9 (52.9%)	0.283
Clinical remission at 24 months (*n* = 39)	34 (87.2%)	20 (87.0%)	14 (87.5%)	0.960
BVAS 0 at 6 months (*n* = 40)	40 (100%)	23 (100%)	17 (100%)	1.000
BVAS 0 at 12 months (*n* = 40)	40 (100%)	23 (100%)	17 (100%)	1.000
BVAS 0 at 24 months (*n* = 39)	39 (100%)	23 (100%)	16 (100%)	1.000
Clinical relapse	5 (12.5%)	4 (17.4%)	1 (5.9%)	0.277
Minor relapse	0	0	0	
Major relapse	5 (12.5%)	4 (17.4%)	1 (5.9%)	
Time to clinical relapse from clinical remission (months)	6.1 (3.1–14.3)	7.9 (4.4–16.7)	2.3	0.157
Change in eGFR, mL/min/1.73 m^2^ (baseline to 12 months)	+ 8 (4–13)	+ 9 (5–13)	+ 6 (0–12)	0.258
Change in eGFR, mL/min/1.73 m^2^ (baseline to 24 months)	+ 10 (3–16)	+ 13 (5–17)	+ 3 (0–13)	0.103
ESKD	1 (2.5%)	0	1 (5.9%)	0.766
Infection requiring hospitalization	5 (12.5%)	3 (13.0%)	2 (11.8%)	0.904
Death	0	0	0	
Observation period (months)	29 (26–32)	28 (25–32)	29 (26–40)	0.652

Data are presented as numbers (%) or medians (interquartile ranges)

Changes in eGFR were analyzed in patients with renal involvement

**Abbreviation**s: eGFR, estimated glomerular filtration rate; ESKD, end-stage kidney disease

During a median observation period of 29 months (IQR 26–32), five patients (12.5%) experienced clinical relapse: four in the continuation group and one in the discontinuation group (Supplemental Table 2).

**Fig. 1:**
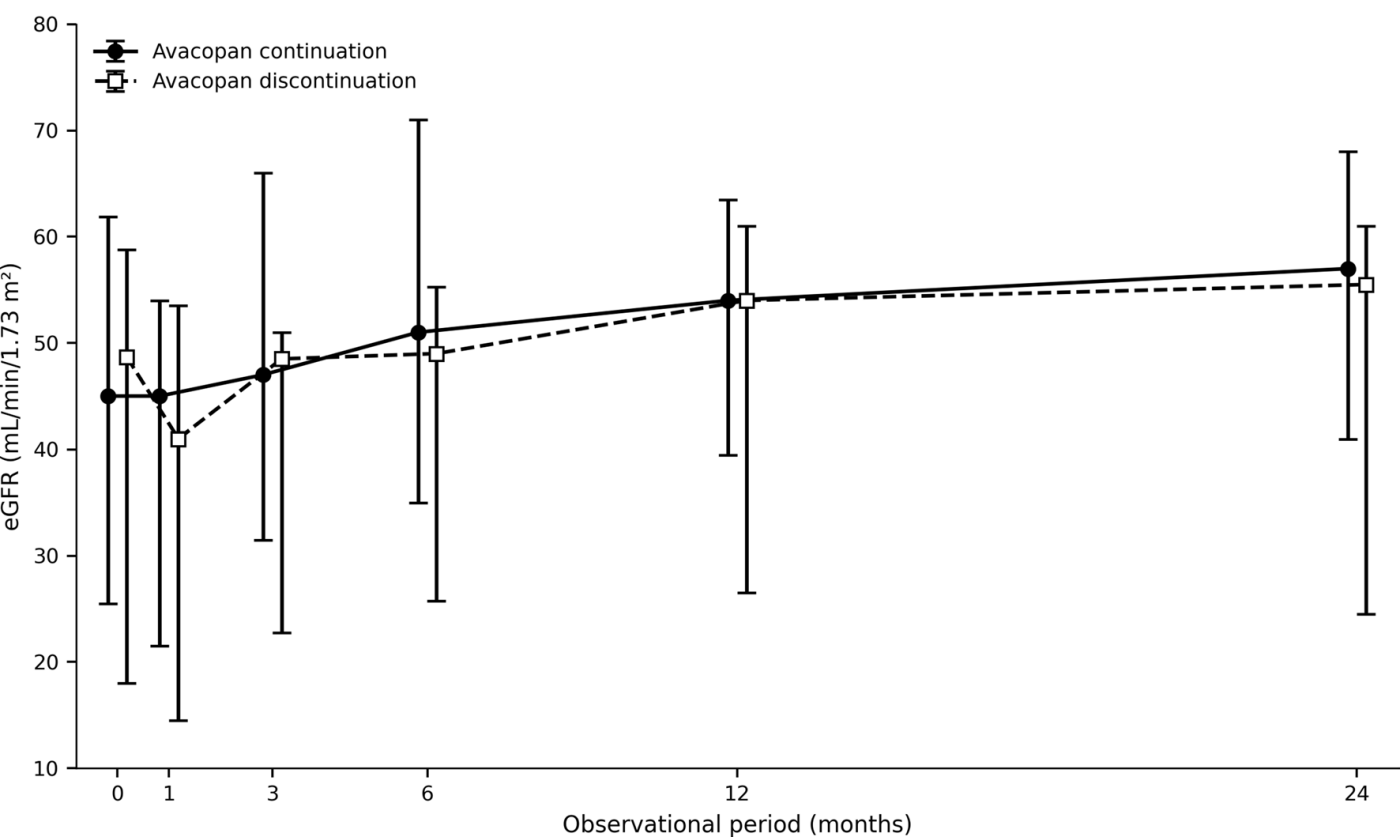
Longitudinal changes in eGFR in patients with renal involvement (*n* = 25). Data are shown as median (IQR). The avacopan continuation group is represented by the solid line with closed circles, and the avacopan discontinuation group by the dashed line with open squares. The x-axis is scaled according to the actual follow-up time points

Among patients with renal involvement, median improvement in eGFR from baseline to 12 and 24 months was + 8 (4–13) and + 10 (3–16) mL/min/1.73 m², respectively, and did not differ significantly between the continuation and discontinuation groups (Fig. 1). One patient (2.5%) in the discontinuation group progressed to ESKD, and no deaths occurred during follow-up. Infection requiring hospitalization occurred in 12.5% of patients, with similar frequencies in the continuation and discontinuation groups (13.0% vs. 11.8%, *P* = 0.904).

Five patients (21.7%) in the continuation group subsequently maintained clinical remission on avacopan monotherapy for a median of 24 months (IQR 16–27) after withdrawal of glucocorticoids and all concomitant non-glucocorticoid immunosuppressive agents (Supplemental Table 1).

### Adverse events related to avacopan

TEAEs attributed to avacopan are detailed in Table 4. Avacopan-related TEAEs were observed in 21 of 40 patients (52.5%). The median time from avacopan initiation to TEAE onset was 1.4 months and was similar between groups. Avacopan was discontinued in all 17 patients in the discontinuation group, and all discontinuations were judged by the treating physicians to be directly related to TEAEs. No discontinuations were attributed to clinical remission, insufficient efficacy, patient preference, or other reasons. In contrast, four patients in the continuation group experienced TEAEs but continued avacopan at a reduced dose. None of the patients in the discontinuation group resumed avacopan during follow-up.

**Table 4:** Adverse events due to avacopan

	All (*n* = 40)	Continuation (*n* = 23)	Discontinuation (*n* = 17)	*P*-value
**TEAEs associated with avacopan**	21 (52.5%)	4 (17.4%)	17 (100%)	< 0.001
Elevated liver enzymes	12 (30.0%)	3 (13.0%)	9 (52.9%)	
Grade 2 (> 3.0–5.0× ULN)	10 (25.0%)	3 (13.0%)	7 (41.2%)	
Grade 3 (> 5.0–20.0× ULN)	2 (5.0%)	0	2 (11.8%)	
Grade 4 (> 20.0× ULN)	0	0	0	
Peak AST level, U/L (ULN, 30)	93 (75–131)	70 (68–92)	116 (56–136)	0.096
Peak ALT level, U/L (ULN, 42)	127 (101–129)	127 (86–128)	126 (102–133)	0.711
Diarrhea/nausea	7 (17.5%)	1 (4.4%)	6 (35.3%)	
Fatigue	2 (5.0%)	0	2 (11.8%)	
**Time to TEAEs from avacopan initiation (months)**	1.4 (0.9–2.1)	1.4 (0.7–1.7)	1.4 (0.9–2.4)	0.687
**Reduced dose after TEAEs and continued avacopan use**	4 (10.0%)	4 (17.4%)	0	
**40 mg/day**	3 (7.5%)	3 (13.0%)	0	
**20 mg/day**	1 (2.5%)	1 (4.4%)	0	

Data are presented as numbers (%) or medians (interquartile ranges)

**Abbreviations:** TEAEs, treatment-emergent adverse events; ULN, upper limit of normal

The most common TEAEs were elevations in liver enzymes (30.0% overall), which were more frequent in the discontinuation group than in the continuation group (52.9% vs. 13.0%). Hepatic events involved AST or ALT levels exceeding three times the upper limit of normal. According to CTCAE version 5.0, liver enzyme elevations were classified as Grade 2 in 10 patients (10/40 [25.0%]; continuation group, 3/23 [13.0%]; discontinuation group, 7/17 [41.2%]) and Grade 3 in 2 patients (2/40 [5.0%]; continuation group, 0/23 [0%]; discontinuation group, 2/17 [11.8%]). No Grade 4 or 5 hepatic events were observed. In all cases, hepatobiliary imaging showed no abnormalities, and viral causes were excluded based on serological and virological testing (negative HAV-IgM, HBV-DNA, HCV antibody, and cytomegalovirus antigenemia).

Gastrointestinal symptoms, such as diarrhea and nausea, occurred in 17.5% of patients (35.3% vs. 4.4% in the discontinuation and continuation groups, respectively), whereas fatigue was reported in two patients (5.0%), exclusively in the discontinuation group. After avacopan discontinuation, liver enzyme elevations normalized within 2 weeks despite continued use of concomitant medications, including trimethoprim-sulfamethoxazole, vitamin D, and gastric acid-suppressive agents. Gastrointestinal symptoms also resolved after avacopan discontinuation or dose reduction. Fatigue improved after avacopan discontinuation in both affected patients and was therefore considered a TEAE by the treating physicians.

Four patients (17.4%) in the continuation group maintained avacopan therapy at a reduced dose after TEAEs: three patients with hepatotoxicity (20 mg/day in one and 40 mg/day in two) and one patient with gastrointestinal intolerance (40 mg/day). In all four patients, symptoms and laboratory abnormalities improved after dose reduction, and clinical remission was maintained without relapse, with successful glucocorticoid discontinuation during follow-up.

## Discussion

This study provides real-world data on clinical outcomes in patients with AAV followed for more than 1 year after avacopan initiation. Such evidence remains limited, and our findings add to this understudied area. In our previous report [10], which evaluated outcomes during the first 12 months after treatment initiation, discontinuation due to adverse events, particularly hepatotoxicity, was relatively common; however, remission rates, relapse rates, renal outcomes, and glucocorticoid tapering were comparable between patients who continued and those who discontinued avacopan. With a larger sample size and longer follow-up than in our previous report, the overall clinical outcomes beyond the first year were broadly similar to those observed during the first year.

A recent systematic review [16], which focused exclusively on real-world avacopan studies and included our previous 12-month report, provides useful context for interpreting the present extended follow-up study. That review reported high 6-month remission rates in routine clinical practice. In the present cohort, all evaluable patients achieved BVAS = 0 at 6, 12, and 24 months. However, the proportion achieving our stricter definition of clinical remission, which required both BVAS = 0 and complete glucocorticoid withdrawal, was 47.5% at 6 months and subsequently increased to 62.5% and 87.2% at 12 and 24 months, respectively. This finding suggests that the lower early clinical remission rate in our cohort mainly reflected the glucocorticoid withdrawal requirement and tapering strategy, rather than persistent vasculitis activity.

In the present cohort, five patients in the continuation group remained in remission on avacopan monotherapy after withdrawal of glucocorticoids and all concomitant non-glucocorticoid immunosuppressive agents, including RTX and conventional agents (Supplemental Table 1). These patients shared several clinical characteristics, such as a favorable initial response to induction therapy, achievement of ANCA negativity, and absence of subsequent ANCA elevation during follow-up.

Although this observation is exploratory and based on a small number of patients, it raises the possibility that, in selected patients with adequately controlled inflammatory activity during induction therapy, remission may be maintained with avacopan monotherapy. This is consistent with emerging reports and discussions regarding treatment de-escalation, maintenance strategies, and avacopan monotherapy in AAV [23–31]. Further studies are required to clarify which patients can safely transition to monotherapy and for how long avacopan should be continued as a single agent.

Adverse events related to avacopan, particularly elevated liver enzymes and gastrointestinal symptoms, were a major reason for treatment discontinuation in this cohort. The same systematic review also noted that hepatotoxicity among TEAEs appeared to be more frequently reported in Japanese cohorts [16]. Consistent with this observation, liver enzyme elevations were observed in 30.0% of patients in our cohort and were more frequent in the discontinuation group. The frequency of liver enzyme elevations in our cohort appeared higher than that reported in randomized trials, which may reflect differences in patient characteristics, concomitant medications, monitoring practices, and population-specific susceptibility in real-world settings. In our predominantly elderly Japanese cohort with MPO-ANCA-positive disease, older age, lower body weight, comorbidities, polypharmacy, and possible population-specific pharmacogenetic differences affecting CYP3A4-mediated drug metabolism may have contributed to the observed toxicity [32–36]. Experimental studies also suggest that C5a–C5aR signaling contributes to hepatocyte regeneration and liver repair, raising the possibility that C5aR blockade might influence recovery from liver injury; however, the clinical relevance of this mechanism in avacopan-treated patients remains uncertain [37]. These mechanisms remain speculative, and causal inference is limited by the retrospective design. These findings highlight the importance of careful safety monitoring in routine practice, especially in older patients with multiple comorbidities.

In addition, in our cohort, dose reduction allowed continuation of avacopan in selected patients who developed TEAEs, including hepatotoxicity and gastrointestinal intolerance, with improvement after dose reduction. None of these patients experienced relapse during follow-up. These findings are exploratory in nature, and the optimal dose of avacopan and appropriate indications for dose reduction remain unclear. At present, no validated tools are available to determine the minimum effective and safe dose for individual patients, highlighting an important area for future research.

At our institution, avacopan was generally continued beyond 12 months unless treatment-emergent adverse events occurred, with the aim of minimizing concomitant immunosuppressive therapy whenever possible. In clinical practice, continuation was considered particularly when further glucocorticoid tapering was desired, when reduction of other immunosuppressive agents was planned, when relapse risk was considered non-negligible, and when avacopan was well tolerated, especially in older patients in whom balancing disease control against treatment-related toxicity was important. However, this approach reflected our institutional practice rather than an established standard of care, and our findings should not be interpreted as supporting routine prolonged avacopan therapy in all patients with AAV who have achieved remission. Taken together, our findings suggest that avacopan may be a useful glucocorticoid-sparing option in selected patients with AAV in routine clinical practice. Its optimal duration and place in maintenance therapy, however, remain uncertain and should be determined in future prospective studies.

This study has some limitations. It was a single-center, retrospective analysis with a relatively small sample size. The duration of avacopan therapy, decisions regarding dose reduction, and reasons for discontinuation were not determined by a standardized protocol. Because patients were classified according to whether avacopan was eventually continued or discontinued after treatment initiation, survivor/immortal time bias cannot be excluded. Therefore, comparisons of outcomes between groups should be interpreted as descriptive rather than causal. ANCA titers and complement activation markers were not measured at predefined intervals, limiting the ability to correlate immunological changes with clinical outcomes, and complement activation markers are not routinely measured in clinical practice to guide therapy in AAV. Furthermore, the high proportion of older patients with multiple comorbidities may limit the generalizability of our findings. Larger prospective studies are needed to better define the optimal use and duration of avacopan therapy in AAV.

In conclusion, in this retrospective single-center cohort, clinical outcomes beyond 1 year appeared broadly comparable between patients who continued avacopan and those who discontinued treatment because of adverse events. Further prospective studies are needed to define the optimal use, patient selection, and duration of avacopan therapy in AAV.

## Supplementary Information

Below is the link to the electronic supplementary material.

Supplementary Material 1: Characteristics of patients who maintained avacopan monotherapy after discontinuation of all other immunosuppressive agents.

Supplementary Material 2: Clinical characteristics of patients who experienced relapse.

## Data Availability

The data that support the findings of this study are available on request from the corresponding author. The data are not publicly available due to privacy or ethical restrictions.

## References

[1] Jennette JC, Falk RJ. Small-vessel vasculitis. N Engl J Med. 1997;337:1512–23. 10.1056/nejm199711203372106. 9366584 10.1056/NEJM199711203372106

[2] Kallenberg CGM. Pathogenesis of ANCA-associated vasculitis. Clin Exp Rheumatol. 2015;33:S11–4. 26457917

[3] Kudo T, Nakazawa D, Watanabe-Kusunoki K, Kanda M, Shiratori-Aso S, Abe N, et al. Regulation of NETosis and inflammation by cyclophilin D in myeloperoxidase-positive antineutrophil cytoplasmic antibody-associated vasculitis. Arthritis Rheumatol. 2023;75:71–83. 10.1002/art.42314. 35905194 10.1002/art.42314

[4] Yang JJ, Pendergraft WF, Alcorta DA, Nachman PH, Hogan SL, Thomas RP, et al. Circumvention of normal constraints on granule protein gene expression in peripheral blood neutrophils and monocytes of patients with antineutrophil cytoplasmic autoantibody-associated glomerulonephritis. J Am Soc Nephrol. 2004;15:2103–14. 10.1097/01.asn.0000135058.46193.72. 15284296 10.1097/01.ASN.0000135058.46193.72

[5] Schanzenbacher J, Hendrika Kähler K, Mesler E, Marcel Karsten C, Leonard Seiler D. The role of C5a receptors in autoimmunity. Immunobiology. 2023;228:152413. 10.1016/j.imbio.2023.152413. 37598588 10.1016/j.imbio.2023.152413

[6] Shiratori-Aso S, Nakazawa D. The involvement of NETs in ANCA-associated vasculitis. Front Immunol. 2023;14:1261151. 10.3389/fimmu.2023.1261151. 37781373 10.3389/fimmu.2023.1261151 PMC10539550

[7] Stone JH, Merkel PA, Spiera R, Seo P, Langford CA, Hoffman GS, et al. Rituximab versus cyclophosphamide for ANCA-associated vasculitis. N Engl J Med. 2010;363:221–32. 10.1056/nejmoa0909905. 20647199 10.1056/NEJMoa0909905 PMC3137658

[8] Jayne DRW, Merkel PA, Schall TJ, Bekker P, ADVOCATE Study Group. Avacopan for the treatment of ANCA-associated vasculitis. N Engl J Med. 2021;384:599–609. 10.1056/NEJMoa2023386. 33596356 10.1056/NEJMoa2023386

[9] Harigai M, Kaname S, Tamura N, Dobashi H, Kubono S, Yoshida T. Efficacy and safety of avacopan in Japanese patients with antineutrophil cytoplasmic antibody-associated vasculitis: a subanalysis of a randomized phase 3 study. Mod Rheumatol. 2023;33(2):338–45. 10.1093/mr/roac037. 35482532 10.1093/mr/roac037

[10] Tagami R, Yamaguchi M, Sugiyama H, Kinashi H, Imai K, Kamiya K, et al. Real-world effectiveness and safety of avacopan in AAV. BMC Rheumatol. 2025;9:8. 10.1186/s41927-025-00456-4. 39844309 10.1186/s41927-025-00456-4 PMC11756139

[11] Ushio Y, Shimada H, Wakiya R, Nakashima S, Miyagi T, Sugihara K, et al. Avacopan is effective in inducing remission for MPA/GPA, regardless of changes in serum C5a levels: a single-center study in Japan. BMC Rheumatol. 2025;9:99. 10.1186/s41927-025-00555-2. 40790248 10.1186/s41927-025-00555-2 PMC12337394

[12] Zonozi R, Aqeel F, Le D, Cortazar FB, Thaker J, Zabala Ramirez MJ, et al. Real-world experience with avacopan in antineutrophil cytoplasmic autoantibody-associated vasculitis. Kidney Int Rep. 2024;9:1783–91. 10.1016/j.ekir.2024.03.022. 38899183 10.1016/j.ekir.2024.03.022 PMC11184253

[13] Zimmermann J, Sonnemann J, Jabs WJ, Schönermarck U, Vielhauer V, Bieringer M, et al. Avacopan in anti-neutrophil cytoplasmic autoantibodies-associated vasculitis in a real-world setting. Kidney Int Rep. 2024;9:2803–8. 10.1016/j.ekir.2024.07.007. 39291204 10.1016/j.ekir.2024.07.007 PMC11403094

[14] Draibe J, Espigol-Frigolé G, Cid MC, Prados MC, Guillén E, Villacorta J, et al. The real-world use and effectiveness of avacopan in routine practice for the treatment of ANCA vasculitis. First experiences in Spain. Rheumatology (Oxford). 2025;64:2019–26. 10.1093/rheumatology/keae534. 39352795 10.1093/rheumatology/keae534

[15] Gabilan C, Belliere J, Moranne O, Pfirmann P, Samson M, Delattre V, et al. Avacopan for anti-neutrophil cytoplasm antibodies-associated vasculitis: a multicentre real-world study. Rheumatology (Oxford). 2025;64:2214–9. 10.1093/rheumatology/keae359. 39001799 10.1093/rheumatology/keae359

[16] Berke I, Keller F, Untersulzner C, Shin JI, Park PG, Oh SS, Callemeyn J, Kronbichler A. Systematic Review of Efficacy and Safety of Avacopan in Real-World Clinical Practice. Kidney Int Rep. 2025;11(4):103753. 10.1016/j.ekir.2025.103753. 41726008 10.1016/j.ekir.2025.103753 PMC12917383

[17] Yamaguchi S, Yamazaki M, Kido T, Hounoki H, Muraishi N, Tajiri K, et al. A case of vanishing bile duct syndrome during treatment of microscopic polyangiitis with avacopan. Rheumatology (Oxford). 2024;63:e120–2. 10.1093/rheumatology/kead285. 37307092 10.1093/rheumatology/kead285

[18] Kojima K, Fukui S, Tanigawa M, Sumiyoshi R, Koga T, Shimakura A, et al. Severe prolonged liver abnormality with jaundice during treatment for granulomatosis with polyangiitis with rituximab and avacopan. Rheumatology (Oxford). 2024;63:e101–3. 10.1093/rheumatology/kead509. 37740250 10.1093/rheumatology/kead509

[19] Watts R, Lane S, Hanslik T, Hauser T, Hellmich B, Koldingsnes W, et al. Development and validation of a consensus methodology for the classification of the ANCA-associated vasculitides and polyarteritis nodosa for epidemiological studies. Ann Rheum Dis. 2007;66:222–7. 10.1136/ard.2006.054593. 16901958 10.1136/ard.2006.054593 PMC1798520

[20] Flossmann O, Bacon P, de Groot K, Jayne D, Rasmussen N, Seo P, et al. Development of comprehensive disease assessment in systemic vasculitis. Ann Rheum Dis. 2007;66(3):283–92. 10.1136/ard.2005.051078. 16728460 10.1136/ard.2005.051078 PMC1855994

[21] Matsuo S, Imai E, Horio M, Yasuda Y, Tomita K, Nitta K, et al. Revised equations for estimated GFR from serum creatinine in Japan. Am J Kidney Dis. 2009;53:982–92. 10.1053/j.ajkd.2008.12.034. 19339088 10.1053/j.ajkd.2008.12.034

[22] Arnold S, Kitching AR, Witko-Sarsat V, et al. Myeloperoxidase-specific antineutrophil cytoplasmic antibody-associated vasculitis. Lancet Rheumatol. 2024;6(5):e300–13. 10.1016/S2665-9913(24)00025-0. 38574743 10.1016/S2665-9913(24)00025-0

[23] Moiseev S, Lee JM, Zykova A, Bulanov N, Novikov P, Gitel E, et al. The alternative complement pathway in ANCA-associated vasculitis: further evidence and a meta-analysis. Clin Exp Immunol. 2020;202:394–402. 10.1111/cei.13498. 32691878 10.1111/cei.13498 PMC7670131

[24] Miyake H, Tanabe K, Yamaji S, Kihara T. Early transition to avacopan from glucocorticoids applied during induction therapy for microscopic polyangiitis with rapidly progressive glomerulonephritis. CEN Case Rep. 2024;13:277–83. 10.1007/s13730-023-00841-3. 38093149 10.1007/s13730-023-00841-3 PMC11294286

[25] Hellmich B, Sanchez-Alamo B, Schirmer JH, Berti A, Blockmans D, Cid MC, et al. EULAR recommendations for the management of ANCA-associated vasculitis: 2022 update. Ann Rheum Dis. 2024;83:30–47. 10.1136/ard-2022-223764. 36927642 10.1136/ard-2022-223764

[26] Zonozi R, Cortazar FB, Jeyabalan A, Sauvage G, Nithagon P, Huizenga NR, et al. Maintenance of remission of ANCA vasculitis by rituximab based on B cell repopulation versus serological flare: A randomised trial. Ann Rheum Dis. 2024;83:351–9. 10.1136/ard-2023-224489. 38123922 10.1136/ard-2023-224489

[27] Chalkia A, Jayne D. ANCA-associated vasculitis—treatment standard. Nephrol Dial Transpl. 2024;39:944–55. 10.1093/ndt/gfad237. 10.1093/ndt/gfad237 PMC11210069 37947275

[28] Roccatello D, Padoan R, Sciascia S, Iorio L, Nic An Ríogh E, Little MA. Might maintenance therapy be discontinued once clinical remission is achieved in ANCA-associated vasculitis? Autoimmun Rev. 2024;23:103438. 10.1016/j.autrev.2023.103438. 37652397 10.1016/j.autrev.2023.103438

[29] Alberici F, Flossmann O, Lamprecht P, Loudon KW, Padoan R, Popov T, et al. Antineutrophil cytoplasmic antibody-associated vasculitis: insights into relapse risk and future management directions. Front Immunol. 2025;16:1655326. 10.3389/fimmu.2025.1655326. 41050707 10.3389/fimmu.2025.1655326 PMC12489576

[30] Ubara Y, Oba Y, Kurihara S, Sekine A, Yamanouchi M, Hasegawa E, et al. A case of renal limited myeloperoxidase anti-neutrophil cytoplasmic antibody-positive vasculitis treated with maintenance avacopan monotherapy. CEN Case Rep. 2025;14:85–9. 10.1007/s13730-024-00910-1. 38955948 10.1007/s13730-024-00910-1 PMC11785830

[31] Kubota S, Hanai S, Tanaka-Mabuchi N, Ito R, Nakagomi D. Is it possible to use avacopan alone in the induction of remission in ANCA-associated vasculitis? Rheumatol Adv Pract. 2024;8:rkae100. 10.1093/rap/rkae100. 39233788 10.1093/rap/rkae100 PMC11374021

[32] Hishida E, Nagata D. Avacopan-associated Liver Injury in Japanese Patients with ANCA-associated Vasculitis: Clinical, Genetic, and Mechanistic Perspectives. Intern Med. 2026;65(7):941–2. 10.2169/internalmedicine.6273-25. 40930832 10.2169/internalmedicine.6273-25 PMC13106723

[33] Mori K, Shirai T, Mutoh T, Inoue J, Fujishima F, Kubo S, et al. Drug-induced liver injury related to avacopan therapy. Rheumatology. 2025;64:2533–40. 10.1093/rheumatology/keae689. 39672792 10.1093/rheumatology/keae689

[34] Yamashita R, Izumi Y, Takane K, Kinoshita A, Hiramoto J. Drug-induced liver injury due to avacopan improved by mycophenolate mofetil: A case report. Med (Baltim). 2025;104:e42121. 10.1097/MD.0000000000042121. 10.1097/MD.0000000000042121 PMC11999426 40228281

[35] Li Y, Meng A, Wang Y. The safety profiles of avacopan on microscopic polyangiitis and granulomatosis with polyangiitis: a real-world pharmacovigilance analysis. Front Immunol. 2025;16:1654735. 10.3389/fimmu.2025.1654735. 41132645 10.3389/fimmu.2025.1654735 PMC12540178

[36] Liang G, Liu X, Gao M, Yang B, Song Y, Liu Q. A real-world disproportionality analysis of avacopan in anti-neutrophil cytoplasmic antibodies associated vasculitis: insights from FDA adverse event reporting system. Pharmacol Res Perspect. 2025;13:e70194. 10.1002/prp2.70194. 41243819 10.1002/prp2.70194 PMC12620843

[37] Strey CW, Markiewski M, Mastellos D, Tudoran R, Spruce LA, Greenbaum LE, Lambris JD. The proinflammatory mediators C3a and C5a are essential for liver regeneration. J Exp Med. 2003;198(6):913–23. 10.1084/jem.20030374. 12975457 10.1084/jem.20030374 PMC2194207

