# Supplementary material for: Real-world outcomes of avacopan beyond the first year in antineutrophil cytoplasmic antibody-associated vasculitis: a retrospective cohort study
Source: BMC Rheumatol. 2026 May 19;10:58. doi: 10.1186/s41927-026-00655-7 (PMC13353023; doi:10.1186/s41927-026-00655-7)
Supplement: Supplementary file 1 — Supplementary Material 1: Characteristics of patients who maintained avacopan monotherapy after discontinuation of all other immunosuppressive agents. [file 41927_2026_655_MOESM1_ESM.docx]

**Supplemental Table 1**

**Characteristics of patients who maintained avacopan monotherapy after discontinuation of all other immunosuppressive agents**

| Patient | Age (years) | Sex | AAV subtype | Disease status (new/relapse) | ANCA type | Baseline BVAS | Organ involvement | Induction therapy | Concomitant immunosuppressive therapy during avacopan monotherapy | Time to glucocorticoid withdrawal (months) | Duration of avacopan monotherapy (months) | Observation period (months) | Clinical relapse during follow-up | ANCA-negative conversion | TEAE during monotherapy | Management of TEAE |
| --- | --- | --- | --- | --- | --- | --- | --- | --- | --- | --- | --- | --- | --- | --- | --- | --- |
| 1 | 81 | M | MPA | New | MPO | 9 | Lung | GC＋RTX | No | 10.1 | 19.1 | 29.2 | No | Yes | No | – |
| 2 | 80 | F | MPA | New | MPO | 14 | Lung / Nerve / Cutaneous | GC＋RTX | No | 4.6 | 26.7 | 31.3 | No | Yes | Liver enzyme elevation | Reduced dose of avacopan (60 → 40 mg/day) |
| 3 | 61 | F | MPA | New | MPO | 12 | Nerve / Kidney | GC＋RTX | No | 16.0 | 24.3 | 40.3 | No | Yes | No | – |
| 4 | 83 | F | MPA | New | MPO | 20 | Lung / Nerve / Kidney | GC＋RTX | No | 13.2 | 27.0 | 40.2 | No | Yes | No | – |
| 5 | 89 | M | MPA | New | MPO | 18 | Lung / Kidney | GC　monotherapy | No | 10.8 | 13.7 | 24.5 | No | Yes | No | – |

Concomitant immunosuppressive therapy included glucocorticoids and all non-glucocorticoid immunosuppressive agents, including rituximab and conventional immunosuppressive agents.

**Abbreviations: AAV, anti-neutrophil cytoplasmic antibody–associated vasculitis; ANCA, anti-neutrophil cytoplasmic antibody; BVAS, Birmingham Vasculitis Activity Score; GC, glucocorticoid; MPA, microscopic polyangiitis; MPO, myeloperoxidase; RTX, rituximab; TEAE, treatment-emergent adverse event.**
