# Supplementary material for: Real-world outcomes of avacopan beyond the first year in antineutrophil cytoplasmic antibody-associated vasculitis: a retrospective cohort study
Source: BMC Rheumatol. 2026 May 19;10:58. doi: 10.1186/s41927-026-00655-7 (PMC13353023; doi:10.1186/s41927-026-00655-7)
Supplement: Supplementary file 2 — Supplementary Material 2: Clinical characteristics of patients who experienced relapse. [file 41927_2026_655_MOESM2_ESM.docx]

**Supplemental Table 2**

**Characteristics of patients who experienced relapse**

| **Patient** | **Age (years)** | **Sex** | **AAV subtype** | **Baseline BVAS** | **Organ involvement at diagnosis** | **Induction therapy** | **Maintenance therapy** | **Time from induction to relapse (months)** | **Avacopan status at relapse** | **Avacopan dose at relapse (mg/day)** | **Time from avacopan discontinuation to relapse (months)** | **Prednisolone dose (mg/day) at relapse** | **Organ involved at relapse** | **Treatment after relapse** | **Outcome** |
| --- | --- | --- | --- | --- | --- | --- | --- | --- | --- | --- | --- | --- | --- | --- | --- |
| **1** | **73** | **F** | **MPA** | **11** | **Pulmonary/ENT/** **Nervous system** | **GC＋RTX** | **RTX** | **18.9** | **Continued** | **60mg** | **-** | **2** | **Nervous system** | **GC increased** | **Remission** |
| **2** | **80** | **M** | **MPA** | **18** | **Pulmonary/Kidney** | **GC＋RTX** | **RTX** | **3.8** | **Continued** | **60mg** | **-** | **5** | **Interstitial pneumonia** | **GC increased** | **Remission** |
| **3** | **45** | **M** | **MPA** | **8** | **Nervous system** | **GC＋RTX** | **RTX** | **6.2** | **Continued** | **60mg** | **-** | **2.5** | **Diffuse alveolar hemorrhage** | **GC increased** | **Remission** |
| **4** | **79** | **F** | **MPA** | **18** | **Pulmonary/Kidney** | **GC＋RTX** | **RTX** | **9.8** | **Continued** | **60mg** | **-** | **7.5** | **Interstitial pneumonia** | **GC increased** | **Remission** |
| **5** | **70** | **F** | **MPA** | **22** | **Pulmonary/Kidney/Nervous system** | **GC＋RTX** | **RTX** | **2.3** | **Discontinued due to TEAE** | **-** | **1.9** | **7.5** | **Nervous system** | **GC increased** | **Remission** |

**Abbreviations: AAV, anti-neutrophil cytoplasmic antibody–associated vasculitis; ANCA, anti-neutrophil cytoplasmic antibody; BVAS, Birmingham Vasculitis Activity Score; GC, glucocorticoid; MPA, microscopic polyangiitis; MPO, myeloperoxidase; RTX, rituximab; TEAE, treatment-emergent adverse event.**
